# Real-world data in patients with *BRCA* mutated breast cancer treated with poly (ADP-ribose) polymerase inhibitors

**DOI:** 10.3332/ecancer.2023.1633

**Published:** 2023-11-21

**Authors:** Evelyn Lilian Beas-Lozano, Haydeé Cristina Verduzco-Aguirre, Roberto Gonzalez-Salazar, Yanin Chavarri-Guerra

**Affiliations:** Department of Hematology and Oncology, Instituto Nacional de Ciencias Médicas y Nutrición Salvador Zubirán, Mexico City 14630, Mexico

**Keywords:** breast cancer, BRCA1 gene, BRCA2 gene, poly(ADP-ribose) polymerase inhibitors

## Abstract

Breast cancer is the most common type of cancer globally. Hereditary breast cancer accounts for 10% of new cases and 4%–5% of cases are associated to pathogenic variants in *BRCA1* or *BRCA2* genes. In recent years, poly-adenosine-diphosphate-ribose polymerase inhibitors (PARPi) olaparib and talazoparib have been approved for patients with *BRCA*-associated, HER2 -negative breast cancer. These drugs have shown positive results in the early and advanced setting with a favourable toxicity profile based on the OlympiAD, OlympiA and EMBRACA phase 3 trials. However, patients included in these randomised trials are highly selected, making toxicity and efficacy in patients encountered in routine clinical care a concern. Since the approval of olaparib and talazoparib for advanced human epidermal growth factor receptor 2-negative (HER2-negative) breast cancer, several phase IIIb–IV trials, expanded access cohorts, and retrospective cohorts have provided information on the efficacy and tolerability of these treatments in patient subgroups underrepresented in the registration trials, such as older adults, patients with poor performance status, and heavily pretreated patients. The aim of this review is to present a critical review of the information regarding the use of PARPi in real-world breast cancer patients.

## Background

Breast cancer is the most common malignant tumour globally, with an incidence of 47.8 cases per 100,000 habitants reported worldwide in 2020 [1]. Incidence and mortality vary among countries, in part due to access to optimal screening programs and novel therapies, as well as family history, environmental and ethnic factors [2, 3]. Although mortality rates have improved in the last 20 years, a contrast in survival persists between women living in high and low-middle income countries. As informed by the CONCORD cohort, 5-year survival in women diagnosed from 2005 to 2009 was greater than 80% in high-income countries, while survival was lower than 70% in low-middle income countries [4], highlighting the benefit of opportune diagnosis and access to better care. This was exemplified during 2010–2014, where 5-year survival for breast cancer in high-income countries such as Australia and the United States of America (USA) was 89.5% and 90.2%, respectively, in stark contrast to countries with a low income, with survival as low as 66.1% in India [5].

Hereditary breast cancer accounts for 10% of new breast cancer cases and 4%–5% are associated with pathogenic variants (PVs) in a high-penetrance gene, transmitted in an autosomal dominant fashion [3]. *BRCA1* was first identified in 1990 on chromosome 17 by Hall *et al* [6] using analyses in families with suggestive pedigrees. Subsequently, *BRCA2* was identified in chromosome 13 in 1994 [7]. Both genes express proteins that are crucial for deoxy nucleotide acid (DNA) double-strand repair. Although *BRCA1* and *BRCA2* PVs are inherited in an autosomal dominant fashion, a biallelic inactivation is required for its pathogenic phenotype [8]. This results in an elevated lifetime risk of breast cancer, with up to 80% for *BRCA1* mutation carriers and 60% for *BRCA2*, compared to a lifetime risk of 12.9% in women in general population [9, 10]. Importantly, tumours associated with germline PVs in *BRCA1* (*gBRCA1m*) tend to have higher histologic grades and are more likely to yield triple-negative tumours (58.1%) compared to tumours associated with germline PVs in *BRCA2* (*gBRCA2m*) and noncarriers (34.4% and 7.5%, respectively) [9]. Nevertheless, it should be noted that this does not necessarily result in a worse prognosis. An analysis of 16 studies including 10,180 patients revealed that the presence of* BRCA* PVs did not lead to a worse overall survival (OS) rate (HR 1.06, 95% CI 0.84–1.34, *p* = 0.61). This conclusion was also supported when examining the impact of *BRCA1* and *BRCA2* PVs separately on OS (*BRCA1*: HR 1.20, 95% CI 0.89–1.61, *p* = 0.24; *BRCA2*: HR 1.01, 95% CI 0.80–1.27, *p* = 0.95) [11]. This might be a consequence of a higher sensitivity to drugs that cause DNA damage, such as platinum agents [12]. Hence, it was only after the advent of poly-adenosine-diphosphate-ribose polymerase inhibitors (PARPi) that this specific population had a chance for targeted treatment.

**Figure 1.**
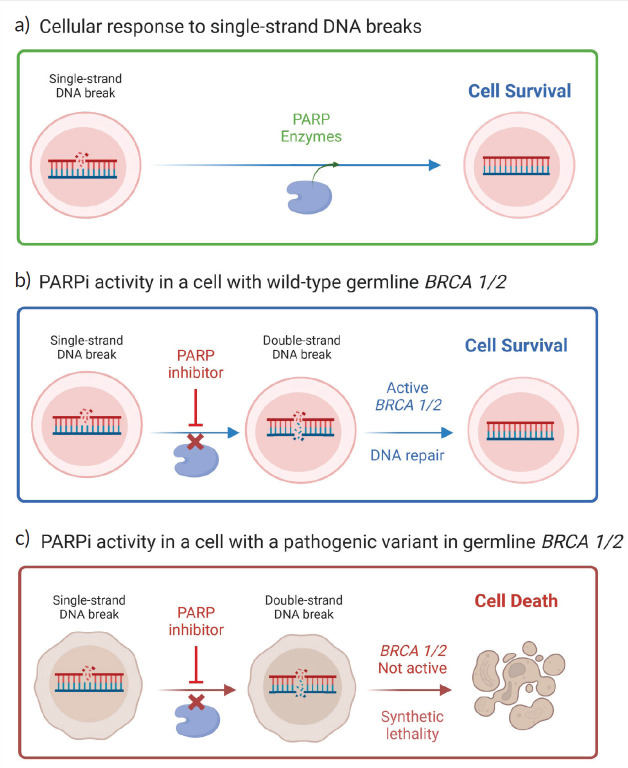
PARPi mechanism of action. (a): In healthy cells, the PARP enzymes play a key role in the repair of single-strand breaks because they recognise the damaged DNA site, bind to it and recruit other repair enzymes to the site of break. (b): By administering PARPi, the cell is unable to repair single-strand breaks due to a collapse of the replication fork which leads to double-strand breaks. In cells with wild-type germline BRCA (and other HRR -related genes), double-strand breaks are repaired via the HRR pathway. (c). In BRCA mutated cells, the HRR pathway is compromised and these double-strand breaks are not susceptible to repair which leads to eventual cell death. Adapted from ‘PARPi: Treatment for BRCA Mutant Breast Cancer’, by BioRender.com (2023). Retrieved from https://app.biorender.com/biorender-templates.

Based on the principle of synthetic lethality, PARPi hinders DNA double-strand repair (Figure 1). DNA damage response pathways target damage in either single or double-strand breaks. The three major DNA damage response pathways for single-strand breaks are base excision repair (BER), nucleotide excision repair and mismatch repair, while the two major pathways for double-strand breaks are homologous recombination repair (HRR) and non-homologous end joining (NHEJ) [13]. PARP enzymes play a key role in the BER pathway. They act by recognising the damaged site in DNA, binding to it, and catalysing the transfer of adenosine-diphosphate-ribose (ADP-ribose) units from NAD+ to itself, creating a polymer called poly(ADP-ribose) that can recruit and activate other repair enzymes to the site of the break. This process is known as PARylation [14]. PARP1 is a constitutively active enzyme that is always present in the cell and is involved in the initial detection and signalling of DNA damage. PARP2, on the other hand, is a more specialised enzyme that is activated only in response to specific types of DNA damage and plays a role in the later stages of repair [15]. Upon PARPi administration, single-strand breaks are not repaired, leading to the collapse of replication forks, leading to the generation of double-strand breaks [16]. In healthy cells, HRR would play a fundamental role in repairing the former. However, in *BRCAm* cells, HRR is compromised, and thus these double-strand breaks are not susceptible to repair. This results in an increase in genomic instability, which leads to eventual tumour cell death due to the activation of the remaining and imperfect NHEJ repair pathway [17].

Four PARPi are approved for the treatment of *BRCA*-associated cancers. However, only two PARPi have been approved for the treatment of breast cancer: talazoparib and olaparib. Talazoparib showed benefit in the metastatic setting based on the EMBRACA phase 3 trial, while olaparib has been approved in the adjuvant and metastatic setting based on the phase 3 OlympiA and OlympiAD trials, respectively [18–20]. Patients included in these trials were required to carry a deleterious *gBRCA1/2m* detected by BRACAnalysis by Myriad Genetics, Food Drug Administration (FDA)-approved test with high sensitivity and specificity [21]. However, PARPi have proven to be effective in a wider range of patients; those with somatic *BRCA1/2m*, homologous recombination deficiency (HRD) or mutations in HRR-related genes. This has been specially studied in the treatment of advanced ovarian cancer [22–25]. These alterations are not uncommon in breast cancer patients, especially in the triple negative subtype, with a reported incidence of HRD as high as 69% in a systematic review [26].

Almost 5 years have passed since the first approval of PARPi in breast cancer patients and real-world data has emerged regarding its use. This review offers an evaluation of the information available on this subject.

### PARPi in the metastatic setting

Olaparib and talazoparib currently are the only PARPi approved for patients with hormone receptor-positive/HER2-negative advanced breast cancer and a *BRCA1* or *BRCA2* mutation detected by germline sequencing. Table 1 includes demographic and clinical characteristics of the four trials in the metastatic setting that showed benefit with PARPi therapy.

In the OlympiAD trial, patients with metastatic breast cancer (MBC) who had received no more than two previous chemotherapy regimens were randomly assigned to receive olaparib or single-agent chemotherapy (capecitabine, eribulin or vinorelbine). Progression-free survival (PFS), the primary endpoint, was significantly longer for patients who received olaparib (7.0 months versus 4.2 months, HR 0.58); the response rate was also higher with olaparib (59.9% versus 28.8%). Grade 3+ toxicity was lower in patients who received olaparib (36.6% versus 50.5%) [20]. On longer follow-up, however, no statistically significant OS improvement was observed (19.3 months versus 17.1 months, HR. 0.90) [27], although the trial was not powered for this outcome.

Talazoparib was approved in the metastatic setting due to information from the EMBRACA trial, where patients with advanced breast cancer who have received no more than three previous cytotoxic regimens were randomly assigned to talazoparib or chemotherapy (capecitabine, eribulin, gemcitabine or vinorelbine). The primary endpoint of the trial was PFS, which was significantly longer in patients who received talazoparib (8.6 months versus 5.6 months, HR 0.54). Objective response rate (ORR) was higher with talazoparib (62.6% versus 27.2%). Hematologic grade 3–4 events were higher with talazoparib (55% versus 38%), while non-hematologic grade 3 events were lower with talazoparib than with chemotherapy (32% versus 38%) [28]. The final analysis of OS did not show a significant difference between the treatment arms (19.3 months versus 19.5 months, HR 0.84) [18].

Although, in the OlympiAD trial all subtypes of breast cancer (those with HR positive, HER2 negative disease and triple negative) had an improvement in PFS, triple negative breast cancer (TNBC) had the greatest benefit when compared to HR positive tumours: OR 0.56 (95% CI 0.34–0.98) and 0.65 (95% CI 0.47–0.91), respectively. Similarly, in the EMBRACA trial, both subgroups had an improvement in PFS with HR 0.60 (95% CI 0.41–0.87) and HR 0.47 (95% CI 0.32–0.71), respectively. Based on these subgroups analyses in both trials PARPi are recommended in the first-line for TNBC tumours and for endocrine refractory tumours according to the inclusion criteria.

Regarding OS and subgroup analyses, it is important to mention that the final OS in the OlympiAD trial showed a greater benefit among those patients who had not received prior chemotherapy for MBC: first-line treatment 22.6 versus 14.7 months (HR 0.51; 95% CI 0.29–0.90; *p* = 0.02) compared to those with prior chemotherapy 18.8 versus 17.2 months (HR 1.13; 95% CI 0.79–1.64; *p* = NS). One of the factors to take in consideration when interpreting these results is the higher sensitivity to platinum drugs in the *BRCA*m population [29, 30]. In fact, the population that received a previous platinum agent seems to have a greater benefit, HR 0.83 (CI 95% 0.49–1.45) versus HR 0.91 (CI 95% 0.64–1.33). Although it was not statistically significant which might be attributed to a higher proportion of patients receiving subsequent platinum agents in the control arm (42.3% versus 29.3%) [20]. On the other hand, in the EMBRACA trial benefit was not different among patients who received previous regimens of chemotherapy except for the patients who received previous platinum treatment with less benefit in PFS HR 0.76 (95% CI 0.40–1.45) versus 0.59 (95% CI 0.34–1.02). A limitation of both trials was to include platinum-based agents as an option in the standard therapy group to compare its effectivity against platinum therapy which opens the question of the best initial treatment option for patients with *BRCA*m.

Furthermore, other trials have tried to answer the utility of combining platinum and PARPi, the BROCADE3 phase III trial, randomised patients to receive veliparib or placebo in combination with carboplatin and paclitaxel. Results showed a greater benefit in PFS in the veliparib group with 14.5 months versus 12.6 months (*p* = 0.0016), confirming its utility in this patient population [31].

### Real world data on effectiveness of PARPi in metastatic breast cancer

The phase IIIb single-arm study LUCY assessed in a real-world setting, the clinical effectiveness of olaparib in a similar population to that of the OlympiAD trial: the trial enrolled 252 patients with HER2-negative MBC with a documented deleterious or suspected deleterious germline *BRCA* mutation (*gBRCAm*); in a later amendment, patients with somatic *BRCA* mutations were also included, but only 3 patients with this characteristic were enrolled in the study [32]. The median age of the cohort with a *gBRCAm* was 45 years; 52% had hormone receptor-positive disease, while 48% had TNBC. One hundred and thirty-seven patients (54.4%) received olaparib as first-line treatment (defined as no previous chemotherapy for advanced disease). Among patients with hormone receptor-positive disease, 19.1% had received therapy with CDK4/6 inhibitors.

In the final analysis of this trial [33], the median PFS was 8.2 months (95% CI 7.0–9.2) and the median OS was 24.9 months (95% CI 21.1–27.9). Median OS was longer in patients with hormone receptor-positive disease (27.4 months) than in those with hormone receptor-negative disease (21.1 months). OS was also longer in patients who received olaparib as a first-line treatment for MBC (27.4 months) compared to those who received it in the second or third line (22.7 months); no formal statistical comparisons were performed between subgroups, but mirrors what was already seen in OlympiAD.

The OS observed in the LUCY trial was longer than that previously reported in the OlympiAD trial; however, the rate of patients who received olaparib as a first-line treatment (defined as not having received chemotherapy in the advanced/metastatic setting) was higher in the former (54.4%) than in the latter (33.2%). Also, in the OlympiAD trial, patients were required to have previously received an anthracycline and a taxane; in the LUCY study, patients were able to enroll having received only one of these drugs – 86.7% of patients had previously received an anthracycline, while 88.6% had previously received a taxane.

The VITAL phase IV study aimed to ensure the effectiveness and safety of talazoparib in a real-world setting. This study consisted of two cohorts: the first included 86 patients treated through the French Early Access Program (93% with a germline *BRCA1/2* mutation and 7% with a somatic mutation); the second enrolled 85 patients treated according to the European Marketing Approval. The primary endpoint of this study was the time to treatment discontinuation, which was 9.0 months in the first cohort. No difference was observed in time to treatment discontinuation according to hormone receptor status, type of mutation (*BRCA1* versus *BRCA2*), prior treatment with platinum-based chemotherapy or number of previous lines of chemotherapy. In this first cohort, median OS was 25.6 months, 6.3 months longer than that reported in the EMBRACA trial. Interestingly, according to a poster presented by Delphine Loirat at the San Antonio Breast Cancer Symposium 2022 (SABCS 2022, Poster P4-01-20) [34], in the first cohort of the VITAL study, only 16% of patients had not received any previous chemotherapy in the metastatic setting, while 38% of patients in the EMBRACA trial had not received any cytotoxic regimens for advanced breast cancer. Results from the second cohort of VITAL were similar: a median time to treatment discontinuation of 9.1 months; this cohort is not yet mature for OS, as presented by Delphine Loirat at the SABCS 2022 (Poster P4-01-04) [35].

Outcomes for the few patients with somatic *BRCA1/*2 mutations included in the LUCY and VITAL studies have not been reported separately, but there are several reports on clinical benefit with olaparib in patients with somatic or combined germline/somatic *BRCA1/2* alterations [36]. Remarkably, a 40-year-old woman with recurrent metastatic TNBC with lung and brain metastases underwent next-generation sequencing, which reported a large deletion of exon 2 in *BRCA1*, a novel (possibly pathogenic) somatic *BRCA2-STARD13* rearrangement and copy number loss of *RAD51*. The patient was treated with olaparib after resection of the symptomatic cerebral metastatic disease, achieving >38 months of disease-free survival [37]. On the other hand, according to information recently presented by Nusrat Jahan at the SABCS 2022 about the Mayo Clinic’s Experience with PARPi, out of 65 patients with MBC and a *BRCA1/2* mutation, 10 (15%) patients had a somatic mutation in homologous recombination-related genes, 7 of them in *BRCA1/2*. On multivariate analysis, somatic *BRCA1/2* mutations were associated with a shorter time to treatment failure (4 months versus 7–8 months in patients with germline *BRCA1/2* mutations).

While patients with active parenchymal central nervous system or leptomeningeal metastases were excluded from the registration trials of PARPi in breast cancer, olaparib has shown activity in the presence of leptomeningeal carcinomatosis, which usually carries a poor prognosis [38]. Thus, PARPi could represent a better-tolerated option compared to more invasive treatments with limited benefit in this situation, such as intrathecal chemotherapy or radiotherapy.

### Real world data on toxicity and tolerability of PARPi in metastatic breast cancer

In the LUCY trial mentioned above, median total treatment duration was 8.0 months (range 0.2–43.3). Adverse events were observed in 85.1% of participants. Most adverse events were grade 1 or 2, with the most common being nausea, anemia, asthenia, fatigue, and vomiting. Grade 3+ adverse events occurred in 17.6% of participants, and almost exclusively consisted of anemia and neutropenia. The rate of discontinuation of olaparib due to adverse events was 4.3%. No toxic deaths were reported.

Olaparib-related adverse events have also been studied through data mining of the USA FDA -Adverse Event Reporting System [39]. Reports in this system are consistent with the evidence from clinical trials and other cohorts: the most common adverse events reported were anemia, thrombocytopenia, nausea, and decreased appetite. Unexpected significant adverse events reported through this system were interstitial lung disease and renal impairment. The median onset time of reported adverse events was 61 days; most events occurred in the first 3 months since olaparib initiation. Other novel adverse events have been described in case reports of patients with MBC with a *BRCA1/2* mutation, including recurrent episodes of erythema nodosum, which may be managed through dose reductions [40].

A retrospective study aimed to identify patient-related risk factors for the development of anemia during treatment with olaparib. This study included 113 patients with advanced breast or ovarian cancer, of which 7.1% of the cohort corresponded to patients with breast cancer. The incidence of grade 3+ anemia was 32.7%, while 61.1% of patients developed grade 1+ anemia. On multivariable logistic regression analysis, low baseline red blood cell (RBC) count, low baseline hematocrit, low baseline hemoglobin, and *BRCA1/2* mutation were significantly associated with the development of grade 3+ anemia, while high baseline RBC, high baseline hematocrit, high baseline hemoglobin level and *BRCA1/2* mutation were significantly associated with grade 1+ anemia [41].

According to the previously mentioned information presented by D. Loirat *et al* at the SABCS 2022, Cohort 1 of the VITAL study reported at least one adverse event in 83% of patients who received talazoparib: the most common adverse events were anemia (33%), thrombocytopenia (17%), neutropenia (12%), alopecia (9%), asthenia (9%) and nausea (8%). Serious adverse events occurred in 16% of patients, mainly anemia and thrombocytopenia. Dose modifications were required in 27% of patients, temporary interruptions in 42%, and in 6% talazoparib had to be permanently discontinued due to adverse events.

In another retrospective analysis of a disease-specific program in USA, European Union and Israel, patterns of treatment were described for 543 patients with advanced HER2-negative breast cancer and a germline *BRCA1/2* mutation [42]. Seventy percent of patients had hormone receptor+/HER2- disease, while 30% had TNBC. Eighty-five percent of patients had a *BRCA1* mutation, and 57% a *BRCA2* mutation. In this cohort, 79 patients (14.5%) had received a PARPi; the proportion of patients who were treated with PARPi varied between patients with hormone receptor+/HER2- breast cancer (5% in first line, 11% in second line, and 12% in third line) and those with TNBC (18% in first line, 44% in second line, and 36% in third line). This study did not mention the specific PARPi that patients received, and survival was not reported in this analysis. Regarding tolerability, the median of days on treatment with a PARPi was 96 days, lower than for other reported therapies such as non-platinum-based chemotherapy (163 days) or endocrine therapy (493 days). Nausea was the most common adverse event, occurring in 32% of patients receiving a PARPi overall, 44% of those who received it in the first line and 31% of those who received it in the second line. Anemia was the second most common adverse event (24% overall; 12% in the first line, and 29% in second- or third-line therapy). Neutropenia was observed in 16% of patients.

### Ongoing trials

Most of the ongoing trials with PARPi in patients with MBC with a *BRCA1/2* mutation are focusing on combinations with other drugs, such as combining olaparib with immune checkpoint inhibitors such as pembrolizumab (NCT03025035), with trastuzumab for patients with HER2-positive disease (NCT03931551), or with sapacitabine, a DNA damage response inhibitor (NCT03641755). Talazoparib is currently being studied in combination with gedatolisib, a dual PI3K/mTOR inhibitor (NCT03911973). Phase IV studies with olaparib are also ongoing in India (NCT04330040).

### PARPi in early breast cancer

In early breast cancer, OlympiA was a large phase 3 randomised clinical trial testing effectivity of olaparib in patients with *gBRCA1* and* BRCA2* PVs HER2 negative high-risk breast cancer. Patients received neoadjuvant or adjuvant chemotherapy and were randomised to olaparib for a year or placebo. Patients with residual disease after neoadjuvant chemotherapy and TNBC or if hormone receptor positive disease should have a high tumour burden. For those who received adjuvant chemotherapy with TNBC (tumour size >2 cm or positive axillary disease) or hormone receptor-positive disease (and at least 4 positive axillary lymph nodes). At a median follow-up of 2.5 years interim analysis showed and improved invasive disease-free survival in the olaparib group (85.9% versus 77.1%; HR 0.58; *p* < 0.001) as well as in distant disease-free survival (87.5% versus 80.4%; HR 0.57; *p* < 0.0001) compared to the placebo group [19]. In the follow-up analysis OS was also improved with olaparib in comparison to placebo (HR 0.68; *p* 0.009) with 89.8% and 86.4% total deaths at 4 years, respectively. The benefit was consistent in all subgroups of patients (TNBC and hormone receptor-positive disease) [43]. At this time, this is the only trial that has shown benefit in the adjuvant setting.

Although PARPi has not been approved in the neoadjuvant setting, studies have showed activity with a high proportion of pathologic complete responses (pCR) in patients with *gBRCAm*. Neoadjuvant PARPi has shown pCR with monotherapy (53%–57%) or in combination with chemotherapy (48%–55%).

In combination with chemotherapy in the neoadjuvant setting, veliparib was tested in combination with carboplatin-paclitaxel followed by doxorubicin-cyclophosphamide, in both the ISP-Y 2 trial (randomised phase II study) and in BrighTNess (randomised phase 3) [44, 45] In both trials a higher pCR was reported in patients with TNBC in comparison with placebo. However, in none of these two trials a clear impact on the *gBRCA* status was found. On the other hand, in the GeparOLA study, olaparib in combination with paclitaxel followed by epirubicin-cyclophosphamide in HDR deficient tumours (*gBRCAm*, somatic *BRCA* mutation or with high HRD score) didn’t show a higher pCR compared to the control arm [46]. As monotherapy, 6 months of talazoparib before standard neoadjuvant chemotherapy showed 53% of pCR [47]. Olaparib as monotherapy also showed to be active in patients with TNBC in the PETREMAC trial with objective response in 56.3% of patients [48]. With this data the question of less intensive chemotherapy regimens for patients who achieved pCR needs further research [47–49].

### The choice between olaparib versus other adjuvant strategies

Adjuvant capecitabine in patients with TNBC with residual disease after neoadjuvant chemotherapy is recommended by international guidelines since it improves OS [50]. However, the question about what therapy should be recommended in carriers of *gBRCAm* arises with the results of the OlympiA trial, where adjuvant capecitabine was not allowed. Taking in account the information in the metastatic setting, where single PARPi have been superior to single-agent chemotherapy and that in the OlympiAD trial olaparib in the first line of MBC was beneficial, suggests that using PARPi earlier might be beneficial [27, 28, 51]. Although there has not been a formal comparison between capecitabine and PARPi in this scenario, the information in the metastatic setting suggests that PARPi might be a better choice in patients with TNBC and *gBRCAm*.

Patients with TNBC might also be a candidate to receive immunotherapy according to the KEYNOTE-522 and GeparNuevo, where patients with stage II–III received immune check point inhibitors plus chemotherapy showed improved outcomes when compared to chemotherapy alone [52, 53]. However, there is no information comparing both strategies or the addition of a PARPi to immunotherapy in the adjuvant setting. In the metastatic setting combining both drugs have been reported to be an active combination, but it is unknown if it is a better choice than a PARPi alone. Therefore, until that information is available the combination of both drugs has been recommended to be individualised based on residual disease, clinical stage and tolerance to therapy [54].

On the other hand, patients with hormone receptor-positive tumours and high-risk characteristics are also candidates for adjuvant CDK4/6 inhibitor with abemaciclib due to improvement in invasive disease-free survival showed by the monarchE trial, where some patients with *gBRCAm* were included [55]. However, other CDK4/6 inhibitors in the adjuvant setting failed to show benefit and combination with PARPi and CDK4/6 inhibitors safety information is not available [56, 57].

### Ongoing trials in early breast cancer

Further studies are investigating the benefit of PARPi in early breast cancer. The ZEST trial (NCT04915755), started in 2021, is a randomised phase III study that has been enrolling patients with localised breast cancer that is either triple negative or HR +/HER2- with a known *BRCA* tissue mutation (irrespective of *BRCA* status). All patients must have detectable circulating tumour DNA after surgery to be randomised to therapy with niraparib or placebo for up to 3 years. Within these patients, two cohorts (patients with wild-type tissue *BRCA* and mutated tissue *BRCA*) will be analysed. The SUBITO trial (NCT02810743), is a phase III study enrolling patients with stage III breast cancer with *gBRCA1/2m* or *BRCA1-*like copy number profile evaluated on tumour tissue. Patients in the control arm receive 1 year of adjuvant olaparib after completing dose dense neo- or adjuvant chemotherapy, while in the experimental patients receive two cycles of intensified alkylating chemotherapy with autologous peripheral stem cell transplantation. The PARTNER trial also tested olaparib in combination with carboplatin in TNBC or in patients with *gBRCAm* which has reported up-to-date tolerability and safety results, while efficacy results are pending. Due to preliminary results with high pCR, other trials in the neoadjuvant settings are also testing the efficacy of PARPi as monotherapy (NCT03329937) and (NCT03499353).

### Special populations: older women

Based on genetic testing criteria, only a few older women with breast cancer will go through genetic risk assessment as compared to younger women [58]. However, a recent study of 1,035 women over the age of 65 in the USA and Latin America with breast cancer found that a significant percentage (10.4%) carried associated germline mutations. Among them, 37% were positive for *BRCA1* and 42% were positive for *BRCA2* [59]. This raises concern for underrepresentation of these women in pivotal phase 3 trials that gave approval to olaparib and talazoparib (only 7.3% in the OlympiAD trial and 3.1% in the OlympiA trial) [18–20].

One of the remaining queries is tolerability in this specific group of patients. A pooled analysis of 398 patients with ovarian cancer (included in phase 1–2 trials evaluating the use of olaparib) showed no differences in tolerability and toxicity between women younger than 65 years and their older counterparts [60]. While there is no data available regarding differences in adherence between the two approved formulations for olaparib, tablets might be preferred. Four tablets a day can be given instead of 16 capsules to achieve the same therapeutic dose. This might be advantageous in patients with underlying cognitive impairment or limited social support [61].

### Patient eligibility criteria for the use of PARPi

FDA approval of the use of PARPi for the treatment of breast cancer is based on data from three randomised clinical trials that took place during different stages of the disease (Table 2). All three trials have in common that they defined eligible patients by the presence of pathogenic or likely PVs in germline *BRCA1* and/or *BRCA2*.

Although FDA approval in all disease settings is currently limited to patients with *gBRCAm*, some studies have further included patients with somatic *BRCA* mutant (*sBRCAm*) tumours and tumours with HRD. For instance, in an effort to evaluate the clinical effectiveness and safety of olaparib in patients with HER2-negative MBC in a real-world setting, the phase IIIB study, LUCY, enrolled patients with *sBRCAm* tumours in addition to patients with PV in *gBRCA* [62]. Moreover, Talazoparib Beyond *BRCA*, a phase II single-arm trial, evaluated the use of talazoparib in patients with either germline or somatic likely pathogenic or PVs in HR-associated genes excluding *BRCA1/2*. Of 20 patients included, 13 had previously treated advanced HER-2 negative breast cancer, whereas seven patients had other types of cancer. Authors reported an ORR of 31% (CI 9%–61%) and a clinical benefit rate of 54% (CI 21%–81%) in breast cancer patients [63]. Likewise, in the early disease setting the GeparOLA trial included patients with HER2-negative disease with HRD as defined by a high HRD score and/or germline or somatic *BRCA1/2* mutations to be randomised to receive neoadjuvant paclitaxel plus olaparib or paclitaxel plus carboplatin, both followed by epirubicin and cyclophosphamide [49]. Finally, the phase II study TBCRC 048 aimed to assess olaparib response in patients with wild-type germline *BRCA* (*gBRCAwt*) but with *sBRCA* mutations or germline or somatic mutations in the homologous recombination-related genes other than *BRCA1/2*. This study showed that patients with germline mutations in *PALB2* and somatic mutations in *BRCA1/2* had high responses to olaparib (82% for *gPALB2m* and 50% for *sBRCA1/2m*) [64].

All this becomes relevant because the population that could derive benefit from the use of PARPi for the treatment of breast cancer seems to extend beyond the carriers of a PV in *gBRCA1/2*, something similar to what has been found in ovarian cancer [65].

## Conclusion

PARPi represent a novel targeted treatment for patients with *BRCA* associated cancers. Olaparib and talazoparib have been approved for a highly selected group of patients with an acceptable toxicity profile. These drugs have yielded promising results, especially for patients with advanced HER-2 negative breast cancer. Thus, we support routine genetic testing of these patients and the use of olaparib and talazoparib for both HR-positive and TNBC. We also favour its use earlier in the disease since patients without previous chemotherapy seem to benefit the most.

Real-world data studies have reproduced the effectiveness of these drugs and showed how they could be useful in different scenarios, especially those with HRD or HRR-related gene mutations. Encouraging results have been reported in the neoadjuvant setting either in monotherapy or in combination with cytotoxic agents, and its combination with immunotherapy is being explored. Real-world information is crucial to understand the effectiveness of these drugs in typically underrepresented patients in the pivotal trials that led to their approval and opened doors to further research for more extensive approval in breast cancer patients.

## Conflicts of interest

Yanin Chávarri-Guerra: Roche research funding and travel expenses; Novartis travel expenses; INVITAE Speakers’ bureau; Astra Zeneca Advisory role.

Haydeé Cristina Verduzco-Aguirre: AstraZeneca travel expenses.

The rest of the authors declare no conflicts of interest.

## Funding statement

The authors affirm that this paper has not been directly funded nor sponsored.

## Figures and Tables

**Table 1.** Demographic and clinicopathological characteristics among patients included in OlympiaD, EMBRACA, LUCY and VITAL.

	OlympiAD		EMBRACA		LUCY	VITAL
**Phase**	**III**		**III**		**IIIB**	**IV**
Intervention	Olaparib	Chemotherapy	Talazoparib	Chemotherapy	Olaparib	Talazoparib
Median age	44	45	45	50	45	51
Race						
White	65.4%	64.9%	66.2%	75%	69.8%	NR
Asian	32.2%	28.9%	NR		8.3%	NR
Black	NR		NR		0.8%	NR
ECOG performance status						
0	72.2%	63.9%	53.8%	58.3%	73.4%	92%
1	27.8%	36.1%	44.3%	39.6%	24.6%	
2	NI		2.1%	1.4%	1.2%	6%
HR-positive	50.2%	50.5%	54.7%	58.3%	52%	53%
Previous platinum agent	29.3%	26.8%	16%	20.8%	32.1%	31%
PARPi as first-line treatment	33.2%	32%	38%	37.5%	54.4%	49%

HR: hormone receptor; NI: not included; NR: not reported; PARPi: PARP inhibitor

**Table 2.** Phase 3 trials for approved PARPi in breast cancer.

Setting	Trial	Population	Study arms	Primary outcome	Overall survival
Adjuvant	OlympyA	*gBRCAm* high risk HER2 negative localised breast cancer: no pathological complete response after neoadjuvant treatment or adjuvant treatment with primary tumour ≥2 cm or axillary node-positive disease	Olaparib versus placebo for 52 weeks	3-year invasive disease-free survival 85.9% versus 7.1% HR 0.58 CI 0.41–0.82 *p* < 0.001	NR
Locally advanced/metastatic setting	OlympiAD	*gBRCAm* MBC hormone ceptor-positive, HER2-negative or TNBC	Olaparib versus chemotherapy (capecitabine, eribulin or vinorelbine)	PFS 7 months versus 4.2 months HR 0.58 CI 0.43–0.89 *p* < 0.001	19.3 months versus 17.1 months HR 0.90 CI 0.66–1.23 *p* 0.513
Metastatic setting	EMBRACA	*gBRCAm* MBC hormone receptor-positive, HER2-negative or TNBC	Talazoparib versus chemotherapy (capecitabine, eribulin, vinorelbine or gemcitabine)	PFS 8.6 months versus 5.6 months HR 0.54 CI 0.41–0.71 *p* < 0.001	19.3 months versus 19.5 months HR 0.84 CI 0.67–1.07 *p* 0.17

CI: confidence interval; HR: hazard ratio; PFS: progression-free survival; NR: not reached; TNBC: triple negative breast cancer
